# 

**Published:** 1871-01

**Authors:** 


*Obituary.*—Dr. Brewster, formerly Court Dentist of France and Russia, recently died in Versailles, after a residence of thirty seven years in Europe. He was appointed court dentist by Louis Philippe, and received numerous decorations from the crowned heads of the continent, and enjoyed quite a reputation as a skillful operator.

